# Dendritic Cell Mediated Delivery of Plasmid DNA Encoding LAMP/HIV-1 Gag Fusion Immunogen Enhances T Cell Epitope Responses in HLA DR4 Transgenic Mice

**DOI:** 10.1371/journal.pone.0008574

**Published:** 2010-01-05

**Authors:** Gregory G. Simon, Yongli Hu, Asif M. Khan, Jingshi Zhou, Jerome Salmon, Priya R. Chikhlikar, Keun-Ok Jung, Ernesto T. A. Marques, J. Thomas August

**Affiliations:** 1 Department of Pharmacology and Molecular Sciences, The Johns Hopkins University School of Medicine, Baltimore, Maryland, United States of America; 2 Department of Biochemistry, Yong Loo Lin School of Medicine, National University of Singapore, Singapore, Singapore; 3 Department of Medicine, Division of Infectious Diseases, The Johns Hopkins University School of Medicine, Baltimore, Maryland, United States of America; New York University, United States of America

## Abstract

This report describes the identification and bioinformatics analysis of HLA-DR4-restricted HIV-1 Gag epitope peptides, and the application of dendritic cell mediated immunization of DNA plasmid constructs. BALB/c (H-2d) and HLA-DR4 (DRA1*0101, DRB1*0401) transgenic mice were immunized with immature dendritic cells transfected by a recombinant DNA plasmid encoding the lysosome-associated membrane protein-1/HIV-1 Gag (pLAMP/gag) chimera antigen. Three immunization protocols were compared: 1) primary subcutaneous immunization with 1×10^5^ immature dendritic cells transfected by electroporation with the pLAMP/gag DNA plasmid, and a second subcutaneous immunization with the naked pLAMP/gag DNA plasmid; 2) primary immunization as above, and a second subcutaneous immunization with a pool of overlapping peptides spanning the HIV-1 Gag sequence; and 3) immunization twice by subcutaneous injection of the pLAMP/gag DNA plasmid. Primary immunization with pLAMP/gag-transfected dendritic cells elicited the greatest number of peptide specific T-cell responses, as measured by *ex vivo* IFN-γ ELISpot assay, both in BALB/c and HLA-DR4 transgenic mice. The pLAMP/gag-transfected dendritic cells prime and naked DNA boost immunization protocol also resulted in an increased apparent avidity of peptide in the ELISpot assay. Strikingly, 20 of 25 peptide-specific T-cell responses in the HLA-DR4 transgenic mice contained sequences that corresponded, entirely or partially to 18 of the 19 human HLA-DR4 epitopes listed in the HIV molecular immunology database. Selection of the most conserved epitope peptides as vaccine targets was facilitated by analysis of their representation and variability in all reported sequences. These data provide a model system that demonstrates a) the superiority of immunization with dendritic cells transfected with LAMP/gag plasmid DNA, as compared to naked DNA, b) the value of HLA transgenic mice as a model system for the identification and evaluation of epitope-based vaccine strategies, and c) the application of variability analysis across reported sequences in public databases for selection of historically conserved HIV epitopes as vaccine targets.

## Introduction

Dendritic cells (DCs) are professional antigen presenting cells (APCs) which play a critical role in the generation of cellular immune responses. DCs acquire antigen (Ag) in the periphery and, upon activation, migrate to lymph nodes where they orchestrate the generation of Ag-specific CD4^+^ and CD8^+^ T-cell responses [1]–[3]. They are being used extensively as Ag carriers in candidate therapeutic vaccine strategies for a variety of disease states, primarily malignancies, to induce specific T-cell responses with little or no toxicity [4]. Previous studies have been conducted with DC transfected with mRNA or plasmid DNA [5]–[9] . Transfection with mRNA provides robust Ag expression, but mRNA is difficult to produce, is unstable, and expression is transient [10]. The use of plasmid DNA transfection provides a more stable gene material and offers longer expression time, but has a history of poor transfection efficiency with DCs and low levels of Ag expression. However, efficient gene transfer into DCs by electroporation has recently been demonstrated [11]–[13].

A number of studies have demonstrated that immunization with DC transfected with mRNA encoding Ag as a chimera of the lysosomal-associated membrane protein (LAMP-1) and thus targeted to the endosomal/lysosomal MHC class II compartment of DCs, can quantitatively and qualitatively influence the repertoire of epitope-specific T-cell responses [14], [15]. LAMP-1 is constitutively expressed at high levels by all nucleated cells, including immature DCs. DNA vaccines encoding LAMP/HIV-1 gag fusion constructs and delivered by subcutaneous (sc) injection have been shown to elicit enhanced antibody, CD4^+^, and CD8^+^ T-cell responses, and prolonged immunological memory to Gag antigen epitopes [16]–[18]. In this study, we tested the effectiveness of immunization of BALB/c and murine H-2 class II-deficient, HLA-DR4/human CD4 (huCD4) Tg mice with DCs transfected *ex vivo* by electroporation with a LAMP/HIV-1 gag DNA plasmid immunogen. The BALB/c mice provided a comparison with the previous pLAMP/gag naked DNA immunization. The HLA DR4 Tg mice were used to compare the DR4-restricted T-cell peptide determinants identified in this study with those of infected humans. Both immunization protocols included different prime-boost formulations with transfected DCs as well as naked plasmid DNA. Additionally, a bioinformatics approach was used to study the representation and variability of the identified epitopes of all database recorded HIV-1 clade B Gag T-cell epitopes. These data provide insight into the efficacy of different vaccine formulations and delivery, the diversity of Gag HLA DR4 T-cell epitopes, and criteria for selection of Gag sequences as vaccine targets.

## Results

### Immunization with pLAMP/Gag-Electroporated Immature DC Induces Enhanced T-Cell Peptide Epitope Repertoire and Avidity in BALB/c and HLA-DR4 Tg Mice

**Figure 1:**
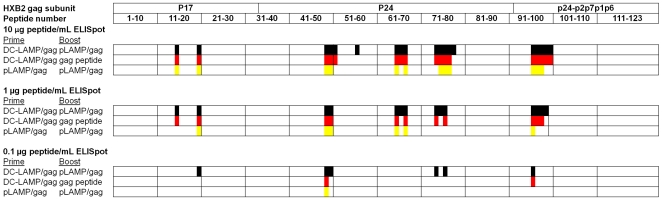
Peptide specificity and relative avidity of IFN-γ-secreting HIV-1 Gag-specific T-cell responses in BALB/c mice immunized with pLAMP/gag-based prime and boost formulations. Mice were immunized subcutaneously with naked pLAMP/gag plasmid 50 µg DNA or with 1×10^5^ pLAMP/gag-transfected immature DCs (DC-LAMP/gag), and boosted with naked pLAMP/gag plasmid (lower and upper lines) or Gag peptides (middle line). Gag peptide-specific T cell responses were measured by *ex vivo* IFN-γ ELISpot assay at peptide concentration of 10 µg/ml (top), 1 µg/ml (middle), and 0.1 µg/ml (bottom), as described in Materials and Methods. The domain structure of the unprocessed HIV-1 Gag protein, and the numbering of the corresponding peptides are indicated on the top. Colored boxes indicate peptides eliciting positive IFN-γ ELISpot responses in mice immunized with pLAMP/gag plasmid DNA prime/boost (yellow), DC-LAMP/gag prime and Gag peptide boost (red), and DC-LAMP/gag prime and pLAMP/gag plasmid DNA boost (black).

We previously have shown that immunization of BALB/c (H-2d) mice with naked DNA encoding pLAMP/gag elicited a broader repertoire and more robust Gag-specific T-cell responses than did immunization with a plasmid expressing the native Gag protein (p55gag) [16]–[20]. In this study, we first compared the T-cell responses of BALB/c mice immunized with immature DCs transfected by pLAMP/gag DNA in comparison to those immunized with naked pLAMP/gag DNA. The peptide-specific T-cell responses were measured by *ex vivo* IFN-γ ELISpot assay with a library of 123 overlapping peptides encompassing the entire p55gag sequence. The relative avidity of the epitope peptides was also evaluated using peptide concentrations varying by two orders of magnitude in the ELISpot assays (10, 1, and 0.1 µg/mL). Immunization with pLAMP/gag-transfected DCs followed by a naked pLAMP/gag boost elicited enhanced IFN-γ secreting T-cell responses with a greater repertoire and apparent avidity than did the naked pLAMP/gag DNA prime and boost immunization, particularly at the lower and intermediate peptide concentrations in the ELISpot assay (Figure 1; Table 1).

**Table 1:** HIV-1 Gag epitope peptides eliciting IFN-γ-secreting T-cell responses in BALB/c (H-2d) mice immunized with pLAMP/gag-based prime and boost formulations^a^.

BEI peptide	HIV Gag peptide positions^b^	Amino acid sequences^b^	IFN-γ SFC/ 10^6^ total splenocytes^c^		
			DC-LAMP/gag, pLAMP/gag	DC-LAMP/gag, Gag peptides	pLAMP/gag, pLAMP/gag
15	57–71	CRQILGQLQPSLQTG	57±12	101±5	40±4
20	77–91	SLYNTVATLYCVHQR	913±14	248±18	652±5
49	193–207	GHQAAMQMLKETINE	1078±25	600±57	748±20
50	197–211	AMQMLKETINEEAAE	929±39	291±17	785±6
51	201–215	LKETINEEAAEWDRL	87±0	158±11	-
56	221–235	GPIAPGQMREPRGSD	62±1	-	-
65	257–271	PVGEIYKRWIILGLN	145±9	54±3	326±19
66	261–275	IYKRWIILGLNKIVR	124±4	82±3	-
67	265–279	WIILGLNKIVRMYSP	99±5	106±19	120±9
74	293–307	FRDYVDRFYKTLRAE	186±8	53±5	-
75	297–311	VDRFYKTLRAEQASQ	233±9	122±5	262±11
76	301–315	YKTLRAEQASQEVKN	138±6	76±4	132±6
77	305–319	RAEQASQEVKNWMTE	89±6	45±3	53±1
78	309–323	ASQEVKNWMTETLLV	46±4	-	-
96	381–395	GNFRNQRKTVKCFNC	245±7	102±3	183±15
97	385–399	NQRKTVKCFNCGKEG	148±8	81±5	48±5
98	389–403	TVKCFNCGKEGHIAK	166±4	122±8	60±6
99	393–407	FNCGKEGHIAKNCRA	128±2	37±3	-
100	397–411	KEGHIAKNCRAPRKK	60±4	31±3	-

a As detected by *ex vivo* IFN-γ ELISpot assay.

b Referring to the HIV-1 HXB2 Gag sequence.

c SFC, spot-forming cells. ELISpot values±SD were obtained from groups of 10 mice immunized with pLAMP/gag-transfected DCs prime and pLAMP/gag plasmid DNA boost (left column), pLAMP/gag-transfected DCs prime and Gag peptides boost (middle column), and pLAMP/gag plasmid DNA prime and boost (right column). Negative results are indicated by dashes.

**Figure 2:**
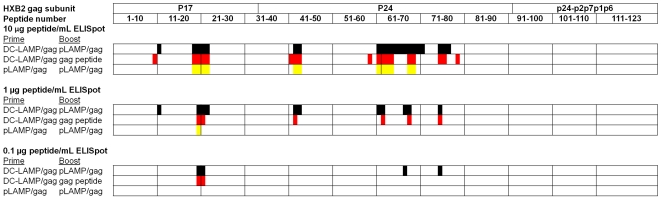
Peptide specificity and relative avidity of IFN-γ-secreting HIV-1 Gag-specific CD4 T-cell responses in HLA-DR4 (DRB1*0401) Tg mice immunized with pLAMP/gag-based formulations. Mice were immunized subcutaneously with 50 µg pLAMP/gag plasmid DNA or with 1×10^5^ pLAMP-1/gag-transfected immature DCs (DC-LAMP/gag), and boosted with pLAMP/gag plasmid DNA (lower and upper lines) or Gag peptides (middle line). Gag peptide-specific CD4 T cell responses were measured by IFN-γ ELISpot assay using CD8-depleted splenocytes, at peptide concentration of 10 µg/ml (top), 1 µg/ml (middle), and 0.1 µg/ml (bottom). The domain structure of the HIV-1 Gag polyprotein is indicated on the top, and the corresponding overlapping peptides are numbered below. Peptides eliciting positive ELISpot responses in mice immunized with pLAMP/gag plasmid DNA prime/boost (yellow), DC-LAMP/gag prime and Gag peptide boost (red), and DC-LAMP/gag prime and plasmid DNA boost (black) are indicated below.

The efficacy of immunization with transfected DCs was further evaluated by immunization of HLA-DR4 Tg mice [21]–[23] with the same immunization protocol as applied to the BALB/c mice. This study had the additional advantage of allowing a comparison of the data from the transgenic animal model to that obtained from studies of infected humans and available in public HIV databases. Splenocytes from the immunized Tg mice were depleted of CD8^+^ T cells and IFN-γ secreting T-cell responses were measured by ELISpot assay as described above. A total of 25 epitope peptides were identified. Similar to the BALB/c study described herein, immunization with pLAMP/gag-transfected DCs prime followed by naked pLAMP/gag boost, or alternatively, pLAMP/gag-transfected DCs prime/Gag peptide boost, elicited a larger number of T-cell responses with greater avidity than did immunization with pLAMP/gag prime/boost (Figure 2; Table 2).

**Table 2:** HIV-1 Gag epitope peptides eliciting CD4 T-cell responses in HLA-DR4 (DRB1*0401) Tg mice immunized with pLAMP/gag-based prime and boost formulations^a^.

BEI peptide number	HIV-1 Gag peptide positions^b^	Amino acid sequences^b^	IFN-γ SFC±SD		
			DC-LAMP/gag pLAMPgag	DC-LAMP/gag gag peptides	pLAMP/gag pLAMP/gag
10	37–51	ASRELERFAVNPGLL	-	69±8	-
11	41–55	LERFAVNPGLLETSE	125±3	-	-
19	73–87	EELRSLYNTVATLYC	165±3	78±16	35±8
20	77–91	SLYNTVATLYCVHQR	225±7	270±2	81±6
21	81–95	TVATLYCVHQRIEVK	380±4	334±28	134±28
22	85–99	LYCVHQRIEVKDTKE	121±20	63±2	53±2
41	161–175	EKAFSPEVIPMFSAL	-	40±11	-
42	165–179	SPEVIPMFSALSEGA	169±7	117±18	62±15
43	169–183	IPMFSALSEGATPQD	120±1	96±1	30±1
59	233–247	GSDIAGTTSTLQEQI	-	42±7	-
61	241–255	STLQEQIGWMTNNPP	130±3	107±3	47±6
62	245–259	EQIGWMTNNPPIPVG	136±8	120±8	50±8
63	249–263	WMTNNPPIPVGEIYK	80±13	63±5	23±5
64	253–267	NPPIPVGEIYKRWII	84±4	44±2	24±6
65	257–271	PVGEIYKRWIILGLN	112±4	-	-
66	261–275	IYKRWIILGLNKIVR	89±2	-	-
67	265–279	WIILGLNKIVRMYSP	302±9	-	-
68	269–283	GLNKIVRMYSPTSIL	99±1	141±2	-
69	273–287	IVRMYSPTSILDIRQ	53±3	43±3	-
70	277–291	YSPTSILDIRQGPKE	29±0	-	-
71	281–295	SILDIRQGPKEPFRD	25±3	-	-
75	297–311	VDRFYKTLRAEQASQ	198±8	182±9	82±18
76	301–315	YKTLRAEQASQEVKN	30±8	58±21	58±21
77	305–319	RAEQASQEVKNWMTE	60±2	-	-
79	313–327	VKNWMTETLLVQNAN	-	55±1	-

a As detected by *ex vivo* IFN-γ ELISpot assay.

b Referring to the HIV-1 HXB2 Gag sequence.

c SFC, spot-forming cells (10^6^ CD8-depleted splenocytes). ELISpot values±SD were obtained from groups of 10 mice immunized with pLAMP/gag-transfected DCs prime and pLAMP/gag plasmid DNA boost (left column), pLAMP/gag-transfected DCs prime and Gag peptides boost (middle column), and pLAMP/gag plasmid DNA prime and boost (right column). Negative results are indicated by dashes.

### Localization and Clustering of the H-2d and HLA-DR4 Epitope Peptides Elicited by pLAMP/Gag Immunization

The ELISpot positive HIV-1 Gag epitope peptides elicited after immunization with pLAMP/gag by the various formulations were present as clusters of overlapping peptides at defined sequence localizations, with the majority of the epitope peptides detected in assays at 10 µg/mL peptide (Figure 2). The T-cell epitope peptides of BALB/c mice were present in four regions of 3 to 5 overlapping sequences comprising 16 of the 19 total epitope peptides (Figure 1; Table 1). The CD4^+^ T-cell epitope peptides of the HLA-DR4 Tg mice were also clustered in 4 to 5 regions detected with ELISpot assays containing 10 µg/mL peptide (Figure 2; Table 2). Results of the different immunization formulations indicated that despite differences between the immunogen delivery as DC, peptides, or naked DNA, there was little difference in the localization of the epitope peptides. DC-mediated immunization enhanced the breath and strength of the T-cell responses to a group of immunogenic peptides that included those elicited by immunization with naked pLAMP/gag, and did not primarly affect the selection of the major immunogenic regions.

### Comparison of Identified HLA-DR4 Epitope Peptides with Reported Human DR4 Epitopes

**Figure 3:**
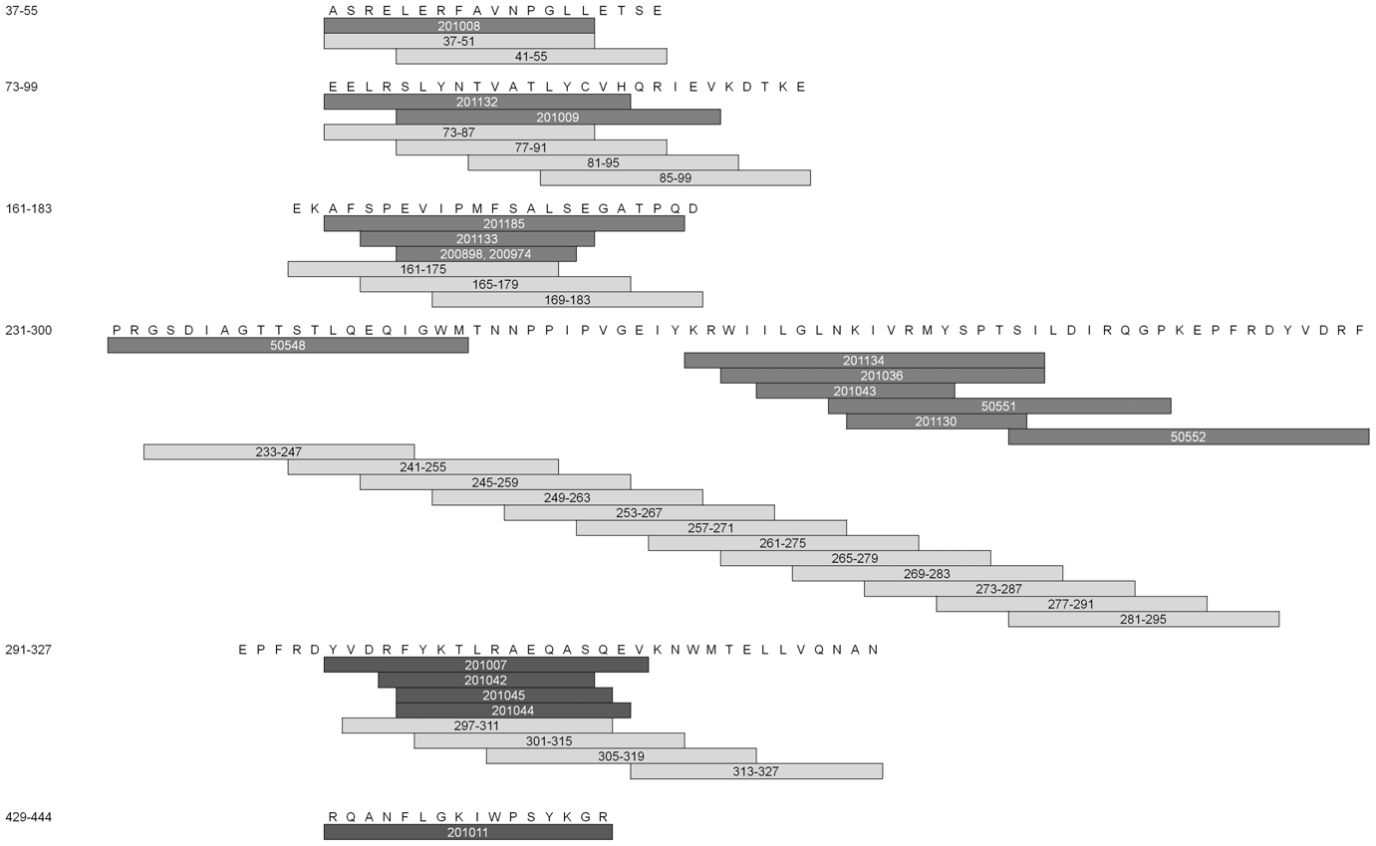
Correlation of known human DR4 CD4 epitopes to ELISpot positive peptides from HLA-DR4 transgenic mice. Previously published CD4^+^ epitopes of HIV infected patients were aligned with IFN-γ ELISpot positive peptide from DR4 mice immunized with LAMP/gag transfected immature DC and plasmid LAMP/gag boost (dark grey bars - known human sequence with HIV molecular immunology database accession number labeled inside, light grey bars - ELISpot positive peptides with the peptide number labeled inside).

The epitopes of the HLA DR4 Tg mice identified in this study were compared to the Gag DR4 epitopes of HIV-1 infected humans reported in the HIV immunology database (Table 3). Strikingly, 21 of the 25 peptides that elicited CD4^+^ T-cell responses in DC immunized HLA-DR4 Tg mice overlapped at least nine consecutive amino acids in 18 of the 19 human HLA-DR4-restricted HIV-1 Gag epitopes (Figure 3). The reported human epitopes were detected by multiple methods, including chromium-release assay, proliferation assay, tetramer binding, intracellular cytokine staining, and IFN-γ ELISpot of CD4 T cells. In addition, the other epitope peptides of this study that are clustered but not coincident with the currently described human CD4 epitopes [peptides no. (position): 62 (245–259), 63 (249–263), 64 (253–267), and 79 (313–327)], suggest the possible presence of other CD4 epitopes. Only one of the reported human epitopes (accession no.: 201011), was not matched by those of the DR4 Tg mice.

**Table 3:** Representation of identified DR4-restricted T-cell epitope peptides and their variants in reported human HIV-1 clade B Gag protein sequences, obtained from the NCBI Entrez protein database^a^.

HLA-DR4-restricted Gag epitope peptide	Total no. of corresponding sequences^b^	Representation of T-cell epitope peptide	Variants of the T-cell epitope peptides			
			Representation of variant peptides	No. of unique variants	% maximum representation of individual variants^c^	Maximum no. of amino acid variations
37-ASRELERFAVNPGLL-51	3282	2322 (∼71%)	960 (∼29%)	162	307 (∼9%)	15
41-LERFAVNPGLLETSE-55	3479	1660 (∼48%)	1819 (∼52%)	266	235 (∼7%)	15
73-EELRSLYNTVATLYC-87	3655	559 (∼15%)	3096 (∼85%)	347	427 (∼12%)	9
77-SLYNTVATLYCVHQR-91	3601	341(∼9%)	3260 (∼91%)	415	459 (∼13%)	6
81-TVATLYCVHQRIEVK-95	3570	144 (∼4%)	3426 (∼96%)	627	311 (∼9%)	8
85-LYCVHQRIEVKDTKE-99	3578	297 (∼8%)	3281 (∼92%)	466	426 (∼12%)	7
161-EKAFSPEVIPMFSAL-175	1792	1343 (∼75%)	449(∼25%)	72	248 (∼14%)	8
165-SPEVIPMFSALSEGA-179	1839	1327 (∼72%)	512 (∼28%)	71	259 (∼14%)	6
169-IPMFSALSEGATPQD-183	1913	1319 (∼69%)	594 (∼31%)	86	267 (∼14%)	8
233-GSDIAGTTSTLQEQI-247	1508	1196 (∼79%)	312 (∼21%)	63	140 (∼9%)	8
241-STLQEQIGWMTNNPP-255	1489	646 (∼43%)	843 (∼57%)	133	151 (∼10%)	10
245-EQIGWMTNNPPIPVG-259	1489	671 (∼45%)	818 (∼55%)	126	165 (∼11%)	13
249-WMTNNPPIPVGEIYK-263	1585	787 (∼50%)	798 (∼50%)	105	228 (∼14%)	14
253-NPPIPVGEIYKRWII-267	1471	1040 (∼71%)	431 (∼29%)	78	134 (∼9%)	6
257-PVGEIYKRWIILGLN-271	1474	958 (∼65%)	516 (∼35%)	76	143 (∼10%)	5
261-IYKRWIILGLNKIVR-275	1473	1014 (∼69%)	459 (∼31%)	66	142 (∼10%)	5
265-WIILGLNKIVRMYSP-279	1421	1013 (∼71%)	408 (∼29%)	53	236 (∼17%)	5
269-GLNKIVRMYSPTSIL-283	1392	790 (∼57%)	602 (∼43%)	51	398 (∼29%)	5
273-IVRMYSPTSILDIRQ-287	1437	542 (∼38%)	895 (∼62%)	73	365 (∼25%)	5
277-YSPTSILDIRQGPKE-291	1460	545 (∼37%)	915 (∼63%)	71	380 (∼26%)	4
281-SILDIRQGPKEPFRD-295	1455	1007 (∼69%)	448 (∼31%)	61	307 (∼21%)	5
297-VDRFYKTLRAEQASQ-311	1221	883 (∼72%)	338 (∼28%)	66	118 (∼10%)	9
301-YKTLRAEQASQEVKN-315	1117	369 (∼33%)	748 (∼67%)	84	409(∼37%)	13
305-RAEQASQEVKNWMTE-319	1113	430 (∼39%)	683 (∼61%)	80	370 (∼33%)	15
313-VKNWMTETLLVQNAN-327	1095	851(∼78%)	244 (∼22%)	64	68 (∼6%)	3

a Example interpretation of the table: The peptide epitope 37-ASRELERFAVNPGLL-51 was present in 2322 sequences (71%) of all 3282 sequences analyzed corresponding to the epitope. The remaining 960 sequences (29%) at that position were variants to the epitope, which were made up of 162 unique peptides with the maximum representation of any of these peptides at 9% (307 peptides) of all the sequences analyzed. The maximum number of amino acid variations between the epitope and the variants was 15 amino acids.

b Total No. of HIV-1 Gag sequences corresponding to the T-cell epitope peptide obtained from the NCBI Entrez protein database (as of August 2008). The number of corresponding sequences for each epitope varies because the database contained both partial and full-length sequences, the consequence of which is that some regions have more sequence information than others.

c For each one of the T-cell epitope peptides, the % minimum representation of individual variants was less than 1%.

### Representation and Variability of the Identified DR4 Epitope Peptide Sequences

Sequences of the 25 DR4-restricted clade B Gag epitope peptides of this study were analyzed for their representation (frequency) and variability in all human HIV-1 clade B Gag sequences listed in the NCBI Entrez protein database (as of August 2008). Many of the epitope peptides were evolutionarily highly variable in their representation in the recorded HIV-1 Gag sequences; 8 were present in 71–79%; 6 in 50 to 69%; and the remaining 11 in less than 50% (Table 3 and Table S1). The number of unique variants for each of the epitope peptides ranged from 51 to a remarkable 627, with as many as 15 amino acid differences per epitope peptide (Table 3). Many of the individual variants for each epitope peptide sequence represented a significant percentage of the total representation ranging, up to 37% (Table S1). However, only four epitopes (out of 25) had a mutant variant sequence as the predominant peptide [peptide no. (position): 21 (81–95), 22 (85–99), 78 (305–319), and 79 (313–327)]; in majority of the cases (21 out of 25), the epitope sequence was the peptide with the highest representation. There was no specific pattern for the distribution of the mutant amino acids in the variants (difference of 3 to 15 aa).

## Discussion

DCs serve critical roles in facilitating immune responses to foreign Ag, in particular by promoting efficient Ag presentation and co-stimulation to CD4^+^ T lymphocytes. Although several different bone marrow−derived cells can present Ag *in vitro*, the main APCs that do so *in vivo* are DCs [24]–[26]. DCs also engage in cross-presentation of peptide epitopes to CD8^+^ T-cells and cross-priming of cells for MHC class I-dependent Ag presentation [27]. Because of these capacities, DC-based vaccines are widely considered to be promising strategies for cancer and persistent pathogen infections such as HIV. This may be compared to naked DNA vaccines which typically express limited amounts of Ag largely restricted to inoculation sites, have restricted access to professional APCs, and poorly activate inflammatory responses, unless combined with additional genetic adjuvants [28]. Here we have studied the immunization of BALB/c (H-2d) and HLA-DR4 Tg mice by autologous DCs transfected *ex vivo* by a DNA plasmid encoding HIV-1 Gag inserted into the LAMP-1 luminal domain, or by direct immunization with the naked DNA plasmid. Immunization by DCs transfected *ex vivo* is believed to deliver a large number of Ag-activated DCs to the immunized animal, with greater antigen presentation to T cells than would occur with direct immunization by the naked DNA vaccine plasmid. However, the transfected DCs as the primary immunogen would likely have limited delivery of antigen to any other cell *in vivo*, greatly reducing the transfection of non-professional APCs and MHC class I dependent presentation of peptide epitopes to CD8^+^ cells [29]. This was likely offset, however, by the subsequent boost with naked DNA encoding LAMP/gag or with peptides, both of which provide Ag access to cells for MHC class I Ag presentation. In contrast to immunization with transfected DC, immunization with the naked DNA plasmid encoding LAMP/gag results in transfection at the site of immunization of large numbers of muscle and other non-professional MHC class I APCs and likely results in relatively large scale activation of CD8^+^ T cells [30]. Targeting of the LAMP/gag chimera to lysosomes of professional APCs is known to result in marked co-localization of the fusion Ag with MHC class II molecules, and activation of both Ag-specific CD4^+^ and CD8^+^ T cells [31], [32]. An additional factor could be the release of HIV Gag from cells as exosome-like vesicles [33] which has been shown to occur in animal model studies [34]. This peculiar property of HIV gag could represent a significant route for its acquisition by DCs *in vivo* in the absence of LAMP-1 targeting.

The epitope-specific T-cell responses to HIV-1 Gag generated by immunization with either the autologous immature DC transfected with pLAMP/gag or naked pLAMP/gag was measured by IFN-γ ELISpot assay using a panel of 123 overlapping Gag peptides encompassing the entire protein. Groups of mice were primed by sc immunization with LAMP/gag DNA-transfected DCs or naked LAMP/gag plasmid DNA, and then boosted with the LAMP/gag plasmid DNA or gag peptide pools. The consistency of T-cell epitope peptide responses elicited by the three immunization protocols with both the BALB/c and HLA DR4 Tg mice is evident from the ELISpot assays with 10 µg/mL peptide. The major differences were the greater fraction of total peptide repertoire and greater apparent avidity of the peptides in the ELISpot assay for either MHC or T-cell receptor binding following the DC LAMP/gag prime-pLAMP/gag boost protocol. The stronger responses to the DC LAMP/gag-pLAMP/gag prime-boost can most simply be attributed to an increased number of antigen presenting DCs following *in vitro* transfection, or to an increased level of antigen presentation or access to MHC loading compartments. Some of the peptides were distinguished by strong T-cell activation at low peptide dose in all tested groups of either the BALB/c or HLA DR4 Tg mice at low peptide dose. Other peptides required higher concentration of peptides in the ELISpot assay and the response was enhanced in or unique to mice primed with plasmid DNA-transfected DCs and boosted with peptides or plasmid DNA. The biological correlates to the differences in the immune responses to the peptides are not known. However, it can be expected that specific epitopes may have unique properties. It has been reported that DCs transfected with interleukin-12 and tumor-associated Ag-encoding mRNA were found to induce high avidity cytotoxic T-lymphocytes (CTLs) [35], and that DCs transfected with RNA encoding tumor antigens resulted in prolonged Ag presentation and the generation of high-affinity tumor-reactive CTLs [36]. Other experiments have also suggested that epitopes that elicit extremely strong T-cell responses may in fact lead to immune tolerance [37].

The low representation of the epitope peptide sequences and their high number of variants represent a major issue for the development of an effective HIV vaccine. The DR4 epitope peptides exhibited variants with as little as 3 or as many as 15 amino acid differences. Vaccine strategies based on Gag or possibly any HIV-1 protein as immunogen may not be effective in preventing natural infection because of the large number of HIV-1 genomic variants as potential altered epitope peptides. Subtle amino acid mutational differences of HLA-restricted T-cell determinants between a vaccine formulation and a natural infection [38] can dramatically alter the magnitude of the T-cell response and result in a wide range of effects from increased immune activation to anergy [39]–[43]. This may possibly be in part overcome by selection of epitope peptides that have high conservation and representation, and where only a minority of subjects would be infected by a virus with a protein sequence that differed from the consensus sequence represented by the vaccine immunogen. It is evident, however, that the extremely high mutation rate of the HIV-1 virus represents a severe problem for the development of an effective vaccine. These issues may be elucidated by further use of HLA transgenic mice for analysis of T-cell activation with selected peptides and their variant forms.

In conclusion, the methodology described in this study, the use of LAMP/gag transfected immature DCs to stimulate an Ag-specific T-cell immune response *in vivo*, provides a broadly applicable strategy for the functional activation of T-cell antigens for HIV therapeutic vaccines that effectively induce HIV-gag specific T-cell responses, with increased avidity and repertoire. The identification of HIV epitopes that represent a large proportion of the circulating virus population must be included to develop a HIV vaccine that can stimulate a robust immune response.

## Materials and Methods

### Cloning of the pLAMP/Gag DNA Construct

The nucleotide sequences of the HIV-1 clade B HXB2 (NCBI Accession No. K03455, M38432) that codes for the p55gag Ag was cloned, with the addition of XhoI and EcoRI restriction sites, into the p43-LAMP-1 eukaryotic expression vector, between the LAMP-1 luminal and transmembrane sequences. The p43-LAMP-1 vector is a pcDNA3.1-based DNA plasmid containing a cytomegalovirus (CMV) promoter, the LAMP-1 luminal sequence, the XhoI and EcoRI cloning sites, the LAMP transmembrane and cytoplasmic sequences, and the bovine growth hormone polyadenylation sequence, flanked by two adeno-associated virus inverted terminal repeat sequences [44].

### Mice

Murine H-2 class II-deficient, HLA-DR4 (DRA1*0101, DRB1*0401)/human CD4 (huCD4) Tg mice [45]–[47] were bred and maintained in the Johns Hopkins University School of Medicine Animal Facility. HLA-DR expression and CD4^+^ T-cell frequency in the experimental mice were evaluated by flow cytometry using HLA-DR-FITC and CD4-PE (BD Bioscience Pharmingen, San Diego, CA) conjugated antibodies, respectively. BALB/c mice were purchased from Charles River (Wilmington, MA). Mice were housed in specific pathogen-free colonies and maintained in a helicobacter-negative mice facility. All animal experiments were performed according to the National Institutes of Health Guide for Care and Use of Experimental Animals and approved by Johns Hopkins Animal Care and Use Committee.

### Bone Marrow Dendritic Cell Culture

Murine DCs were generated from the bone marrow using a protocol slightly modified from Inaba *et al.* [48]. Briefly, the donor mice were euthanized by CO_2_ asphyxiation followed by cervical dislocation, the femurs and tibias collected, and the muscle tissues removed with gauze. The bones were placed in a 60 mm dish with 70% ethyl alcohol for 1 min, washed twice with PBS, and transferred into a fresh dish with RPMI 1640. The bone marrows were then flushed with 3 ml RPMI 1640 per bone and the viable cells were counted using a Vi-CELL Cell Viability Analyzer (Beckman Coulter, Fullerton, CA). The cells were centrifuged at 300g for 10 min, and the supernatant discarded. The cell pellet was resuspended in RPMI-1640 supplemented with 10% mouse serum, 2000 U/ml GM-CSF, 100 units/ml penicillin-streptomycin, 2 mM L-glutamine, 50 µM 2-mercaptoethanol, and 1 M HEPES buffer at 1×10^6^ cells/mL, transferred into a 24-well plate (1ml/well), and incubated in a 37°C incubator with a 5% CO_2_ atmosphere. Fresh medium containing GM-CSF was added every second day. On day seven the immature DCs were harvested by collecting non-adherent and loosely adherent cells.

### Electroporation of Immature Dendritic Cells

After seven days culture, the immature DCs were collected and centrifuged at 300 g for 10 min, resuspended in 100 uL of Nucleofector solution (Lonza Inc, Walkersville, MD) to a final concentration of 2.5×10^5^/100 µL, and 5 µg of the pLAMP/gag plasmid DNA was added to each microcentrifuge tube. The mixture was transferred to an Amaxa certified cuvette and electroporated with the preset Nucleofector program Y-001 for immature DCs. The transfected DCs were then suspended in 400 µl complete media and transferred onto a 48-well plate and incubated overnight in a humidified 37°C 5% CO_2_ incubator.

### DC Immunization Protocol

Three experimental immunization groups of 10 mice (5 pools of 2 mice) was immunized and boosted three to four weeks later by subcutaneous injection at the base of the tail.

Group 1 Prime: 1×10^5^ DCs transfected with p43LAMP/gag plasmid (5 µg); Boost: p43LAMP/gag plasmid (5 µg)

Group 2 Prime: same as Group 1; Boost: pooled peptide (1µg/peptide)

Group 3 Prime: p43LAMP/gag plasmid (50 µg); Boost: same as prime

Group 4 Prime: 1×10^5^ DCs transfected with p43GFP plasmid (5 µg); Boost: p43GFP plasmid (5 µg)

### Splenocyte Isolation

Ten days following the boost immunization, splenocytes were harvested from mice euthanized as previously described. Single cell suspensions were prepared by manual disruption and separation through a 70 µm cell strainer. The cells were washed with complete RPMI (1640 medium supplemented with 10% FBS, 100 units/mL penicillin-streptomycin, 2 mM L-glutamine, 50 µM 2-mercaptoethanol, and 1 M HEPES buffer), centrifuged for 10 min at 300 g at 4°C, and resuspended in 5 ml ammonium chloride for 5 min at room temperature to lyse the red blood cells. Then 25 ml complete RPMI-1640 was added to stop the reaction. The cells were again centrifuged at 300 g at 4°C and the pellet was resuspended in 10 ml complete RPMI-1640. Cells were counted with the Vi-CELL Cell Viability Analyzer and adjusted to a concentration of 1×10^7^/mL in complete RPMI.

### HIV p55gag Synthetic Peptides

A library of 123 overlapping 15 amino acid (aa) peptides spanning the entire HIV-1 HBX2 p55gag protein, more than 80% pure, were obtained from Biodefense & Emerging Infections Research Resources Repository (Manassas, VA). Lyophilized peptides were dissolved at 1 mg/mL in 20% DMSO and water, aliquoted, and stored at −20°C.

### IFN-γ ELISpot Assay

*Ex vivo* IFN-γ ELISpot assays were performed by use of murine IFN-γ ELISpot sets (BD Biosciences) according to manufacturer's protocol. For Tg mice, single cell suspensions from freshly isolated splenocytes in complete RPMI-1640 were depleted of CD8^+^ T-cells using antibody-coated magnetic beads (Miltenyi Biotec. Auburn, CA) according to manufacturer's protocol. A total of 1×10^6^ splenocytes diluted into 100 µl complete RPMI-1640 were added to wells containing individual p55gag peptides. Concanavalin A (Sigma-Aldrich, St. Louis, MO), 5 µg/ml, and culture medium without peptide were used as positive and negative controls, respectively. Following assay protocol, the plates air-dried, and the spots counted using a stereomicroscope at 40× magnification with the ImmunoSpot software (CTL analyzer, Cleveland, OH). The average number of spot forming cells (SFC) was adjusted for 1×10^6^ splenocytes.

### Evaluation Statistics

The average IFN-γ secreting T-cell responses against each peptide for vaccinated mice were compared by a nonparametric *t* test to the background values of control mice primed with a green fluorescent protein expressing p43 plasmid (p43GFP). The responses were considered positive when the *p* value was >0.05 and the average response was >10 IFN-γ SFC/10^6^ splenocytes. Negative control assays for mice immunized with the p43-GFP plasmid DNA commonly gave blank values of 0–10 SFC per 10^6^ splenocytes.

### Comparison of Identified HLA-DR4 Epitope Peptides with Reported Human DR4 Epitopes

The epitopes of the HLA DR4 Tg mice identified in this study were compared to the reported epitopes of HIV-1 infected humans by performing blastp [49] search with an in house database (February 2009) of 388 T-helper/CD4^+^ epitope sequences, obtained from the HIV immunology database. BLAST hit sequences with at least nine consecutive amino acid matches to the query epitope were extracted for further analysis. All identified T-helper/CD4^+^ epitopes in the HIV immunology database were retrieved and records with HLA-DRB1*0401 or DR4 restrictions in the “Species (MHC/HLA)” field were selected.

### Diversity Analysis of the Identified DR4 Epitope Peptide Sequences

A total of 6,403 complete and partial human HIV-1 clade B Gag protein sequences were retrieved from the NCBI Entrez protein database records as of August 2008. Multiple sequence alignment of the sequences was performed by use of Promals3D [50], which was then manually inspected and corrected for misalignments. The diversity of each of the identified HIV-1 Gag DR4 Tg epitope peptide sequence was studied by extracting all the sequences in the alignment corresponding to the position of the epitope, except those that contained gaps (-) or any one of the unresolved characters, including B (asparagine or aspartic acid), J (leucine or Isoleucine), X (unspecified or unknown amino acid) and Z (glutamine or glutamic acid). All the extracted sequences were then analyzed for the representation (frequency) of the epitope sequence and its variants. Variants are defined as peptides with at least one amino acid difference from their corresponding identified epitope.

## Supporting Information

Table S1 The representation of HLA-DR4 transgenic mice epitope peptides and their variants in clade B HIV Gag protein.
(0.35 MB DOC)
