# Supplementary material for: Dendritic Cell Mediated Delivery of Plasmid DNA Encoding LAMP/HIV-1 Gag Fusion Immunogen Enhances T Cell Epitope Responses in HLA DR4 Transgenic Mice
Source: PLoS One. 2010 Jan 5;5(1):e8574. doi: 10.1371/journal.pone.0008574 (PMC2797323; doi:10.1371/journal.pone.0008574)
Supplement: Table S1 — The representation of HLA-DR4 transgenic mice epitope peptides and their variants in clade B HIV Gag protein. (0.35 MB DOC) [file pone.0008574.s001.doc]

**Table S1**. The representation of HLA-DR4 transgenic mice epitope peptides and their variants in reported HIV-1 clade B Gag protein sequence dataset, obtained from the NCBI Entrez protein database.

| Position | HIV Gag epitope peptide and variants**^a^** | Representation in reported sequences of Gag epitope peptide^c^ |
| --- | --- | --- |
| 37-51 | **ASRELERFAVNPGLL** | 2322 (~71%) |
|  | .........L..... | 307 (~9%) |
|  | .........I..... | 101 (~3%) |
|  | ............S.. | 74 (~2%) |
|  | ..K............ | 56 (~2%) |
|  | ...........S... | 36 (~1%) |
|  | ............S.M | 21 (~1%) |
|  | ........SI..... | 15 (<1%) |
|  | .........LD.... | 12 (<1%) |
|  | ..K........S... | 11 (<1%) |
|  | ..........D.... | 10 (<1%) |
|  | 152 other variants^b^ | 317 (<1% each) |
| 41-55 | **LERFAVNPGLLETSE** | 1660 (~48%) |
|  | .............A. | 235 (~7%) |
|  | .....L......... | 234 (~7%) |
|  | ..............G | 214 (~6%) |
|  | ..............D | 129 (~4%) |
|  | .....I......... | 76 (~2%) |
|  | .............AG | 59 (~2%) |
|  | ........S...... | 46 (~1%) |
|  | .......S....... | 43 (~1%) |
|  | ............S.. | 39 (~1%) |
|  | .............P. | 36 (~1%) |
|  | .............AD | 29 (~1%) |
|  | .....L.......A. | 27 (~1%) |
|  | ..............A | 26 (~1%) |
|  | .............T. | 23 (~1%) |
|  | ........S.M.... | 22 (~1%) |
|  | ........A...... | 19 (~1%) |
|  | ..............K | 19 (~1%) |
|  | ........S....A. | 19 (~1%) |
|  | .....L.......P. | 18 (~1%) |
|  | ....SI......... | 17 (<1%) |
|  | .....L........A | 16 (<1%) |
|  | .....L......S.. | 13 (<1%) |
|  | ............SA. | 10 (<1%) |
|  | 243 other variants | 450 ( <1% each) |
| 73-87 | **EELRSLYNTVATLYC** | 559 (~15%) |
|  | ...K..F........ | 427 (~12%) |
|  | ...K........... | 391 (~11%) |
|  | ......F........ | 299 (~8%) |
|  | ...K.......V... | 165 (~5%) |
|  | ...........V... | 119 (~3%) |
|  | ..IK........... | 108 (~3%) |
|  | ...K..F....V... | 93 (~3%) |
|  | ...K.V......... | 89 (~2%) |
|  | .........I..... | 76 (~2%) |
|  | .........I.V... | 75 (~2%) |
|  | ......F....V... | 70 (~2%) |
|  | ...K.....I..... | 69 (~2%) |
|  | ...K..F..I..... | 69 (~2%) |
|  | ......F..I..... | 62 (~2%) |
|  | ........A...... | 60 (~2%) |
|  | ...K.....I.V... | 47 (~1%) |
|  | ......F..I.V... | 38 (~1%) |
|  | ..IK..F........ | 32 (~1%) |
|  | ...K..F.A..V... | 24 (~1%) |
|  | ...K..F..I.V... | 21 (~1%) |
|  | ...K..F.A...... | 21 (~1%) |
|  | ...K..F..I.VI.. | 19 (~1%) |
|  | ..F........V... | 17 (<1%) |
|  | ..F............ | 15 (<1%) |
|  | ..VK........... | 14 (<1%) |
|  | ...K..F..I.V.F. | 13 (<1%) |
|  | ...K..H....V... | 13 (<1%) |
|  | ...K......V.... | 12 (<1%) |
|  | .....VF........ | 12 (<1%) |
|  | .GIK........... | 12 (<1%) |
|  | ...K.VF........ | 12 (<1%) |
|  | ...K..F.AI.V... | 11 (<1%) |
|  | ......F.A..V... | 11 (<1%) |
|  | ...K..H........ | 11 (<1%) |
|  | ...K....A...... | 11 (<1%) |
|  | ..F...F....V... | 10 (<1%) |
|  | ...Q.......V... | 10 (<1%) |
|  | 310 other variants | 538 (<1% each) |
| 77-91 | ..............K | 459 (~13%) |
|  | ..F...........K | 348 (~10%) |
|  | **SLYNTVATLYCVHQR** | 341 (~9%) |
|  | ..F............ | 245 (~7%) |
|  | .......V......K | 155 (~4%) |
|  | .......V....... | 138 (~4%) |
|  | ..F....V....... | 109 (~3%) |
|  | .....I.V....... | 91 (~3%) |
|  | .............E. | 87 (~2%) |
|  | .V............. | 81 (~2%) |
|  | .....I......... | 65 (~2%) |
|  | ..F....V......K | 48 (~1%) |
|  | ..............Q | 48 (~1%) |
|  | ..............G | 47 (~1%) |
|  | ..F..I......... | 43 (~1%) |
|  | ..F..I........G | 41 (~1%) |
|  | ..F...........G | 40 (~1%) |
|  | .....I........G | 38 (~1%) |
|  | .....I.V......K | 38 (~1%) |
|  | ....A.......... | 37 (~1%) |
|  | ..F..I........K | 36 (~1%) |
|  | ..F..........E. | 33 (~1%) |
|  | ..............N | 32 (~1%) |
|  | ..F..I.V....... | 32 (~1%) |
|  | .....I........K | 28 (~1%) |
|  | ..F..........R. | 28 (~1%) |
|  | .............EK | 25 (~1%) |
|  | ..F.A..V....... | 24 (~1%) |
|  | ..F.A.........K | 20 (~1%) |
|  | ..F...........Q | 19 (~1%) |
|  | ..F..I.VI...... | 19 (~1%) |
|  | ..F..I.V......K | 18 (<1%) |
|  | .VF...........K | 17 (<1%) |
|  | .............KK | 16 (<1%) |
|  | ..F..I.V.F..... | 15 (<1%) |
|  | ....A.........N | 15 (<1%) |
|  | ..F...........N | 12 (<1%) |
|  | ..............H | 12 (<1%) |
|  | ..F.AI.V....... | 11 (<1%) |
|  | .............A. | 11 (<1%) |
|  | ....A..V....... | 11 (<1%) |
|  | .......V......G | 11 (<1%) |
|  | ..F..........RK | 11 (<1%) |
|  | ......V........ | 10 (<1%) |
|  | ............QKG | 10 (<1%) |
|  | ..H....V....... | 10 (<1%) |
|  | 370 other variants | 616 (<1% each) |
| 81-95 | ..........K.D.. | 311 (~9%) |
|  | ............D.. | 231 (~6%) |
|  | ..........K.... | 198 (~6%) |
|  | .............I. | 179 (~5%) |
|  | **TVATLYCVHQRIEVK** | 144 (~4%) |
|  | ...V.........I. | 105 (~3%) |
|  | ...V......K.D.. | 84 (~2%) |
|  | ..........K..IR | 81 (~2%) |
|  | ..........K...R | 73 (~2%) |
|  | ..........K..I. | 68 (~2%) |
|  | ...V........... | 63 (~2%) |
|  | ...V......K.... | 55 (~2%) |
|  | .........E..K.A | 43 (~1%) |
|  | .........E..K.T | 42 (~1%) |
|  | .I........G.... | 41 (~1%) |
|  | .I............. | 40 (~1%) |
|  | ............DI. | 40 (~1%) |
|  | .I.V........D.. | 39 (~1%) |
|  | ...V........D.. | 39 (~1%) |
|  | .I..........D.. | 35 (~1%) |
|  | A...........D.. | 35 (~1%) |
|  | .........R..... | 32 (~1%) |
|  | .I........K.... | 25 (~1%) |
|  | ..........K.DIR | 24 (~1%) |
|  | .I.V........... | 24 (~1%) |
|  | ..........G.... | 24 (~1%) |
|  | ..........Q.DIR | 24 (~1%) |
|  | .I........G...R | 23 (~1%) |
|  | A.........K.D.. | 22 (~1%) |
|  | .I.V......K.D.. | 21 (~1%) |
|  | ..........K.D.R | 20 (~1%) |
|  | ..........Q.... | 20 (~1%) |
|  | .I........K.D.. | 19 (~1%) |
|  | .I.V.........I. | 19 (~1%) |
|  | .I.V......K.... | 17 (<1%) |
|  | .I.VI.......D.. | 17 (<1%) |
|  | ..........N.... | 16 (<1%) |
|  | ...V......KV... | 16 (<1%) |
|  | ............DIR | 16 (<1%) |
|  | .I...........I. | 15 (<1%) |
|  | ...V......K..I. | 14 (<1%) |
|  | A..V........D.. | 14 (<1%) |
|  | A.........N.D.. | 14 (<1%) |
|  | ..........G..IR | 13 (<1%) |
|  | ..........H...A | 12 (<1%) |
|  | .I.V.F......D.. | 12 (<1%) |
|  | .I.V........D.R | 11 (<1%) |
|  | ..........K..M. | 11 (<1%) |
|  | .........KK.K.A | 11 (<1%) |
|  | .........EK.K.A | 11 (<1%) |
|  | ..............R | 10 (<1%) |
|  | ..........K.DI. | 10 (<1%) |
|  | ..........Q.K.. | 10 (<1%) |
|  | ..........N..IR | 10 (<1%) |
|  | A..V........... | 10 (<1%) |
|  | ..........G.KI. | 10 (<1%) |
|  | ........QKG...A | 10 (<1%) |
|  | 571 other variants | 1037 (<1% each) |
| 85-99 | ......K.D...... | 426 (~12%) |
|  | ........D...... | 332 (~9%) |
|  | .........I..... | 319 (~9%) |
|  | **LYCVHQRIEVKDTKE** | 297 (~8%) |
|  | ......K........ | 294 (~8%) |
|  | ......K..IR.... | 100 (~3%) |
|  | ......K..I..... | 92 (~3%) |
|  | ......K...R.... | 81 (~2%) |
|  | ......G........ | 79 (~2%) |
|  | ........DI..... | 59 (~2%) |
|  | .....E..K.T.... | 44 (~1%) |
|  | .....E..K.A.... | 43 (~1%) |
|  | ........D....R. | 40 (~1%) |
|  | .....R......... | 39 (~1%) |
|  | ......G...R.... | 32 (~1%) |
|  | ......K.D.R.... | 31 (~1%) |
|  | ........D....Q. | 31 (~1%) |
|  | ......K.D....N. | 31 (~1%) |
|  | ........D.R.... | 30 (~1%) |
|  | ......G.D...... | 24 (~1%) |
|  | ......K.DIR.... | 24 (~1%) |
|  | ......K..M..... | 23 (~1%) |
|  | I.......D...... | 23 (~1%) |
|  | ......Q........ | 23 (~1%) |
|  | ......N........ | 23 (~1%) |
|  | ......N.D...... | 22 (~1%) |
|  | ......K.DI..... | 21 (~1%) |
|  | ......KV....... | 18 (~1%) |
|  | .........IR.... | 18 (~1%) |
|  | ........DIR.... | 18 (~1%) |
|  | ......G..IR.... | 17 (<1%) |
|  | ..........R.... | 17 (<1%) |
|  | ......G..I..... | 14 (<1%) |
|  | .F......D...... | 14 (<1%) |
|  | .....KK.K.A.... | 13 (<1%) |
|  | ......H...A.... | 13 (<1%) |
|  | ........N...... | 12 (<1%) |
|  | ......Q.DIR..T. | 12 (<1%) |
|  | .....EK.K.A.... | 11 (<1%) |
|  | ........GI..... | 11 (<1%) |
|  | .....R..D...... | 11 (<1%) |
|  | ......Q.DIR.... | 11 (<1%) |
|  | ......Q.K...... | 10 (<1%) |
|  | .........I....D | 10 (<1%) |
|  | .....E......... | 10 (<1%) |
|  | ....QKG...A.... | 10 (<1%) |
|  | ........K.A.... | 10 (<1%) |
|  | ......G.KI..... | 10 (<1%) |
|  | 419 other variants | 725 (<1% each) |
| 161-175 | **EKAFSPEVIPMFSAL** | 1343 (~75%) |
|  | ............T.. | 248 (~14%) |
|  | ............A.. | 85 (~5%) |
|  | 70 other variants | 116 (>1% each) |
| 165-179 | **SPEVIPMFSALSEGA** | 1327 (~72%) |
|  | ........T...... | 259 (~14%) |
|  | ........A...... | 88 (~5%) |
|  | ...........A... | 46 (~3%) |
|  | ............D.. | 11 (~1%) |
|  | 67 other variants | 108 (>1% each) |
| 169-183 | **IPMFSALSEGATPQD** | 1319 (~69%) |
|  | ....T.......... | 267 (~14%) |
|  | ....A.......... | 117 (~6%) |
|  | .......A....... | 49 (~3%) |
|  | .............T. | 18 (~1%) |
|  | ........D...... | 11 (~1%) |
|  | 81 other variants | 132 (<1% each) |
| 233-247 | **GSDIAGTTSTLQEQI** | 1196 (~79%) |
|  | .........N..... | 140 (~9%) |
|  | ..............V | 21 (~1%) |
|  | .........S..... | 20 (~1%) |
|  | ......V........ | 15 (~1%) |
|  | 59 other variants | 116 (<1% each) |
| 241-255 | **STLQEQIGWMTNNPP** | 646 (~43%) |
|  | ...........H... | 151 (~10%) |
|  | ...........S... | 147 (~10%) |
|  | .......A....... | 53 (~4%) |
|  | .......A...S... | 53 (~4%) |
|  | .......Q...S... | 36 (~2%) |
|  | .N............. | 26 (~2%) |
|  | .N.....A....... | 23 (~2%) |
|  | .N.........S... | 23 (~2%) |
|  | .N.....A...H... | 21 (~1%) |
|  | .......A...H... | 18 (~1%) |
|  | .......T....... | 16 (~1%) |
|  | .N.........H... | 13 (~1%) |
|  | .......A...G... | 12 (~1%) |
|  | ......V....S... | 12 (~1%) |
|  | ...........S..A | 10 (~1%) |
|  | ...D...A......S | 10 (~1%) |
|  | 3 other variants | 26 (~1% each) |
|  | 114 other variants | 193 (<1% each) |
| 245-259 | **EQIGWMTNNPPIPVG** | 671 (~45%) |
|  | .......H....... | 165 (~11%) |
|  | .......S....... | 157 (~11%) |
|  | ...A........... | 72 (~5%) |
|  | ...A...S....... | 54 (~4%) |
|  | ...A...H....... | 34 (~2%) |
|  | ...Q...S...V... | 31 (~2%) |
|  | ...A...S...V... | 15 (~1%) |
|  | ...T........... | 15 (~1%) |
|  | ..V....S....... | 12 (~1%) |
|  | .......S...V... | 11 (~1%) |
|  | ...A...G....... | 10 (~1%) |
|  | ...A......S.... | 10 (~1%) |
|  | .......S..A.... | 10 (~1%) |
|  | 3 other variants | 25 (~1% each) |
|  | 111 other variants | 199 (<1% each) |
| 249-263 | **WMTNNPPIPVGEIYK** | 787 (~50%) |
|  | ...S........... | 228 (~14%) |
|  | ...H........... | 208 (~13%) |
|  | ...S...V....... | 42 (~3%) |
|  | ...........D... | 39 (~2%) |
|  | ...H.......D... | 19 (~1%) |
|  | ...G........... | 19 (~1%) |
|  | ...S...V...D... | 18 (~1%) |
|  | ...S.......D... | 18 (~1%) |
|  | .......V....... | 12 (~1%) |
|  | ...S..A........ | 11 (~1%) |
|  | ...G...V....... | 11 (~1%) |
|  | ......S........ | 10 (~1%) |
|  | 1 other variant | 8 (~1%) |
|  | 92 other variants | 155 (<1% each) |
| 253-267 | **NPPIPVGEIYKRWII** | 1040 (~71%) |
|  | ...........K... | 134 (~9%) |
|  | ...V........... | 64 (~4%) |
|  | .......D....... | 45 (~3%) |
|  | .......D...G... | 20 (~1%) |
|  | ..S............ | 16 (~1%) |
|  | ..A............ | 14 (~1%) |
|  | ...V...D....... | 14 (~1%) |
|  | 1 other variant | 9 (~1%) |
|  | 70 other variants | 115 (<1% each) |
| 257-271 | **PVGEIYKRWIILGLN** | 958 (~65%) |
|  | .......K...M... | 143 (~10%) |
|  | ...........M... | 119 (~8%) |
|  | ...........I... | 64 (~4%) |
|  | ...D........... | 55 (~4%) |
|  | ...D...G....... | 20 (~1%) |
|  | ...D.......M... | 10 (~1%) |
|  | ..........V.... | 10 (~1%) |
|  | 69 other variants | 95 (<1% each) |
| 261-275 | **IYKRWIILGLNKIVR** | 1014 (~69%) |
|  | ...K...M....... | 142 (~10%) |
|  | .......M....... | 127 (~9%) |
|  | .......I....... | 67 (~5%) |
|  | ...G........... | 23 (~2%) |
|  | ......V........ | 10 (~1%) |
|  | 61 other variants | 90 (<1% each) |
| 265-279 | **WIILGLNKIVRMYSP** | 1013 (~71%) |
|  | ...M........... | 236 (~17%) |
|  | ...I........... | 71 (~5%) |
|  | ............IAL | 18 (~1%) |
|  | ..V............ | 12 (~1%) |
|  | 49 other variants | 71 (<1% each) |
| 269-283 | **GLNKIVRMYSPTSIL** | 790 (~57%) |
|  | ...........V... | 398 (~29%) |
|  | ...........I... | 76 (~5%) |
|  | ...........A... | 26 (~2%) |
|  | ...........S... | 16 (~1%) |
|  | ............N.. | 16 (~1%) |
|  | 1 other variant | 7 (~1%) |
|  | 45 other variants | 63 (<1% each) |
| 273-287 | **IVRMYSPTSILDIRQ** | 542 (~38%) |
|  | .......V....... | 365 (~25%) |
|  | .............K. | 255 (~18%) |
|  | .......I....... | 71 (~5%) |
|  | .......V.....K. | 44 (~3%) |
|  | .......A....... | 20 (~1%) |
|  | ........N...... | 18 (~1%) |
|  | .......S....... | 13 (~1%) |
|  | 1 other variant | 8 (~1%) |
|  | 65 other variants | 101 (<1% each) |
| 277-291 | **YSPTSILDIRQGPKE** | 545 (~37%) |
|  | ...V........... | 380 (~26%) |
|  | .........K..... | 260 (~18%) |
|  | ...I........... | 75 (~5%) |
|  | ...V.....K..... | 42 (~3%) |
|  | ...A........... | 21 (~1%) |
|  | ....N.......... | 17 (~1%) |
|  | ...S........... | 13 (~1%) |
|  | 1 other variant | 8 (~1%) |
|  | 63 other variants | 99 (<1% each) |
| 281-295 | **SILDIRQGPKEPFRD** | 1007 (~69%) |
|  | .....K......... | 307 (~21%) |
|  | ...........S... | 17 (~1%) |
|  | N.............. | 17 (~1%) |
|  | .....G......... | 10 (~1%) |
|  | 1 other variant | 9 (~1%) |
|  | 56 other variants | 88 (<1% each) |
| 297-311 | **VDRFYKTLRAEQASQ** | 883 (~72%) |
|  | .............T. | 118 (~10%) |
|  | ............CT. | 30 (~2%) |
|  | ....F.......... | 29 (~2%) |
|  | ....F........T. | 20 (~2%) |
|  | .....R......... | 18 (~1%) |
|  | ......V........ | 18 (~1%) |
|  | 3 other variants | 23 (~1% each) |
|  | 57 other variants | 82 (<1% each) |
| 301-315 | ...........D... | 409 (~37%) |
|  | **YKTLRAEQASQEVKN** | 369 (~33%) |
|  | .........T..... | 113 (~10%) |
|  | ........CT..... | 31 (~3%) |
|  | ..V........D... | 16 (~1%) |
|  | .R.........D... | 15 (~1%) |
|  | F........T..... | 10 (~1%) |
|  | 6 other variants | 43 (~1% each) |
|  | 72 other variants | 111 (<1% each) |
| 305-319 | **RAEQASQDVKNWMTE** | 430 (~39%) |
|  | .......E....... | 370 (~33%) |
|  | .....T.E....... | 120 (~11%) |
|  | ....CT.E....... | 25 (~2%) |
|  | .......E......D | 17 (~2%) |
|  | .....T.E......D | 13 (~1%) |
|  | 4 other variants | 25 (~1% each) |
|  | 71 other variants | 113 (<1% each) |
| 313-227 | **VKNWMTETLLVQNAN** | 851 (~78%) |
|  | .............S. | 68 (~6%) |
|  | ......D........ | 39 (~4%) |
|  | 4 other variants | 32 (~1% each) |
|  | 58 other variants | 105 (<1% each) |

^a^ The epitope sequences are shown in bold face and the mutations by the respective variant amino acid.

^b^ For variants with representation of less than 10, only the number of such variants and their individual representation is shown.

^c^ Data was collected from the NCBI Entrez protein database (as of August 2008).
